# Metabolic endoscopy and a simplified low-carbohydrate–high-dietary fiber template as novel treatments for hidradenitis suppurativa – A case series

**DOI:** 10.1016/j.jdcr.2023.01.035

**Published:** 2023-02-19

**Authors:** Mandour O. Mandour, Safa Al-Musawi, Esther Idowu, Paul F. Long, Ellie Rashidghamat, Jude A. Oben

**Affiliations:** a Department of Gastroenterology & Hepatology, Guy’s and St Thomas’ Hospital, London, United Kingdom; b Institute of Cancer and Pharmaceutical Science, King’s College, London, United Kingdom; c Department of Dermatology, Guy’s and St Thomas’ Hospital, London, United Kingdom; d Faculty of Life Sciences & Medicine, Guy’s and St Thomas’ Hospital, United Kingdom

**Keywords:** gut-skin axis, hidradenitis suppurativa, low-carbohydrate diet, metabolic endoscopy, obesity, skin microbiome, short -chain fatty acids, BMI, body mass index, HS, hidradenitis suppurativa, IGB, intragastric balloon

## Introduction

Hidradenitis suppurativa (HS) is a chronic inflammatory disease of the skin that typically occurs after puberty and follows a relapsing-remitting course that can significantly affect a patient’s quality of life. It is characterized by painful nodules mainly involving areas of the body with apocrine sweat glands, such as the axillary, inguinal, and anogenital regions. The population prevalence of HS is estimated at 1% to 4% and is strongly associated with obesity and metabolic syndrome.^1^ The disease severity of HS is scored by the Hurley staging system.^2^ Obesity is known to cause several alterations in skin physiology, leading to an increased predisposition to various skin diseases. Not only does obesity affect skin barrier integrity but it also causes alterations in collagen structure and impairment of wound healing because of a reduction in mechanical strength.^3^ Obesity, moreover, is associated with chronic venous disease and lymphoedema by causing microvascular dysfunction.^3^ Patients with obesity are known to have larger intertriginous folds, which lead to increased mechanical friction at flexural sites. This causes injuries at follicular openings, increased levels of proinflammatory cytokines, and sweat retention, leading to skin irritation.^1^, ^2^, ^3^, ^4^, ^5^, ^6^, ^7^ Patients who downsize have been shown to have better clinical outcomes, with a reduction in disease severity and remission.^8^ Unfortunately, many patients with HS who are obese struggle to downsize, and bariatric surgery is not readily available, with waiting lists in some centers upward of 24 months.^9^ Metabolic endoscopy is a growing field aiming to aid weight loss through endoscopic minimally invasive, nonsurgical means. Deployed into the stomach endoscopically and removed after 6 to 12 months, we have previously shown the intragastric balloon (IGB) to be an efficacious nonsurgical modality to aid weight loss in patients who are obese.^10^

## Case series

### Case 1

A woman aged 24 years old with Hurley stage 3 HS had a raised body mass index (BMI) of 36.1 kg/m^2^. She was not on any medications that caused weight gain and had not previously been treated with any medications to aid weight loss. At diagnosis aged 14 years, HS was localized to the axillae with evidence of inflammatory papules, pustules, and draining sinus tracts in both axillae. Her HS, however, later progressed to involve several other regions, including the groin. Axillary and groin swabs grew *Staphylococcus aureus* on multiple occasions with occasional growth of coliforms. She had failed multiple medical treatments for HS, including doxycycline, dapsone, and a combination of clindamycin and rifampicin, and had undergone multiple surgical treatments, including excision from the left axilla and both groins/suprapubic areas, and incision and drainage of the left thigh abscess to treat the HS. Additionally, she had been treated with infliximab 5mg/kg for >12 months with no improvement noted. At the time of referral for consideration of IGB insertion to aid weight loss, her HS had progressed to affect both axillae and bilateral groin regions, and there was a significant disease affecting the buttocks, particularly the perineal area with a sinus tract in the natal cleft. Moreover, there was active pustulation and some evidence of folliculitis affecting the buttocks bilaterally. Her preinsertion weight was 101.3 kg. Six weeks following IGB insertion, the patient had lost 15 kg in weight. The IGB was removed after 6 months, at which point her weight was 92.6 kg, totaling some 8.7 kg (8.6%) weight loss over the period of 6-month. The patient was noted to have made clinical improvement in her HS with a reduction in her HS Hurley stage and markedly improved quality of life. Unfortunately, following the IGB removal, her weight steadily increased and approached her pre-IGB weight with clinically evident worsening in her HS disease.

### Case 2

A 19-year-old woman presented with Hurley stage 1 HS, which initially started aged 11 years with boils on her thighs and progressed to affect her breasts, axilla, and buttocks. Her BMI was 42 kg/m^2^ at the time of referral. She had been previously treated with erythromycin, clindamycin, lymecycline, and isotretinoin, with no improvement in her HS symptoms. She had multiple inflammatory papules and nodules with no evidence of any sinus tract. There were however, some comedones on her thighs. Swabs were taken from the pustules that lightly grew *Staphylococcus lugdunensis*, which was sensitive to flucloxacillin, but resistant to clarithromycin. The patient was started on rifampicin and clindamycin at the time of the first dermatologic clinical assessment. She was struggling to lose weight through exercise, because of the disease burden of recurrent boils afflicting her groin and breasts. She underwent IGB insertion when her weight was 91.8 kg. The IGB was removed after 8 months. At the time of removal, she weighed 76.1 kg (total weight loss at 15.7 kg) equating to 17% weight loss. Two months later, she had lost a further 10 kg in weight, totaling 25.7 kg equating to 28% weight loss. Following this, the patient was reassessed in the HS clinic, where it was noted that her HS was in complete remission.

### Case 3

A 43-year-old woman presented with inflamed nodules to her right axilla with no discharge, active linear ulcers with granulation in the lower portion of the right side of abdomen, groins, suprapubic area, and the natal cleft. Her HS was scored at 3. This had been quiescent for several years on a combination of surgery, rifampicin, clindamycin, and infliximab. She weighed 98.7 kg, a BMI of 41.6 kg/m^2^. She was commenced on a simplified low-carbohydrate template based on Shai et al,^11^ which had shown that a low-carbohydrate calorie nonrestricted diet was superior at inducing weight loss; compared with low-fat calorie-restricted and Mediterranean calorie-restricted diets. Based on this, and with the patients’ help, we designed a patient-friendly, humorous template that was easy to remember –designated as “No-P.” It involved simply avoiding all alcohol and removing foods starting with the letter “P” - potatoes (including sweet potatoes, or derivatives eg, chips or crisps, or yam), pasta, pizza, plantain, pies, pastries (donuts, cakes, and biscuits), puddings (donuts and cakes also), pulses (couscous, lentils, or quinoa), and pilau rice or any form of rice, poppadum’s, panipuri, Peshwari naan (or any form of bread). These “P”-foods are the largest sources of carbohydrate intake among patients in our locality, comprising a high population of Asians, Africans, and Caribbeans, with marked levels of obesity and type 2 diabetes mellitus. Our patient was advised to eat fresh fish, meat, vegetables, and salads plus fruit 3× times a week, to ensure that all macronutrients and micronutrients were eaten. The reduction of fruit to 3 times per week is to reduce the amount of fruit sugar – fructose – because some patients mistakenly eat up to 2 kg of fruit per day. The patient was also advised to exercise some portion control. Following this simplified low-carbohydrate template, the patient was able to lose 7.9 kg (8%) of her initial body weight. At IGB insertion in June 2017, she weighed 90.8 kg. At review in September 2017, she weighed 73 kg, a further weight loss of 17.8 kg (19.6%). Her HS was quiescent with no active inflammatory lesions. A further IGB was inserted in February 2018, at which time she weighed 72.4 kg. When the second IGB was removed in September 2018, she weighed 67.1 kg, a total weight loss of 32%. In September 2020, she weighed 65 kg with her HS still in remission.

## Discussion

HS is a chronic inflammatory skin condition that is strongly associated with obesity. There is mounting evidence to support a reduction in disease severity with weight loss; however, patients with HS and obesity struggle to downsize. Many patients do not meet the criteria for bariatric surgery (BMI >40 or >35 kg/m^2^ with obesity-related comorbidities), and if they do there are often long waiting lists upward of 24 months. In this setting, novel metabolic endoscopic procedures, such as the IGB should be considered.^10^ In a very recent case series of 15 patients with obesity-related hepatic steatosis, we reported that all 15 patients lost ≥10% of initial body weight at 6 months follow-up, after IGB insertion and a significant reduction in carbohydrate consumption. There was also a change in the gut microbiomes of these patients compared with a control group (that also received IGB metabolic endoscopy but who did not change their carbohydrate intake).^12^ Here, we have shown 3 cases where metabolic endoscopy with the IGB gave a clinical improvement in HS through aiding weight loss (summarized in Table I), which had been difficult to achieve through lifestyle modification and medical therapy alone. A very recent report has suggested differences in both the gut and skin microbiota in patients with HS compared with healthy individuals, although a mechanism for chemical communication between the gut-skin axis remains elusive.^13^ Based on our previous studies^10^^,^^12^ and the case series reported here, we propose that the insertion of an IGB concomitant with reduced carbohydrate and high-fiber food intake (the “No-P template”) while promoting weight loss might also revert gut dysbiosis sufficient to improve HS severity. It is well established that the bacteria of the gut microbiome ferment dietary fibers producing short-chain fatty acids that stimulate adipose tissue to upregulate fat oxidation and contribute to weight loss.^14^ HS is characterized by a reduction in sebum production because the pilosebaceous unit is destroyed with fibrosis as the HS progresses.^15^ Reduced sebum has not been investigated as a factor leading to alterations in the skin microbiome associated with HS, although changes in the bacterial community structure of the skin and immune cell infiltration of HS lesions have been well described.^16^^,^^17^

**Table I:** Summary of changes in clinical parameters of the 3 cases following IGB insertion

Clinical parameters	Patient 1	Patient 2	Patient 3∗
Gender	Female	Female	Female
Age, y	24	19	43
Hurley stage at presentation	3	1	3
Initial weight (kg)	101.3	91.8	98.7
Weight following IGB insertion and removal	92.6	76.1	67.1
% Weight loss	8.6	17	32
Hurley stage after removal of IGB	½	Complete remission	Complete remission

*IGB*, Intragastric balloon.

∗ Note that this patient achieved an 8% reduction in weight (from 98.7-90.8 kg) using the “No-P template” before intragastric balloon insertion. The patient continued using the “No-P template” while the intragastric balloon remained *in situ*. Patients attending our service can receive up to 3 metabolic endoscopy treatments. This patient was treated twice, and the weight loss reported is the cumulative weight loss at the end of the second treatment.

We were intrigued to note that of the many microbiome studies of HS few, if any, have focused on the possible role of the fungi (mycobiome) in the pathogenesis of HS. In one of only a few studies, we could find that providing electron micrographic evidence of follicles associated with HS lesions taken from tissues excised following surgery, there are clear images of increased bacteria and immune cell infiltration of the sebaceous glands as expected. Intriguingly, electron-dense structures with a size and shape consistent with yeast cells were distinguishable yet were unreported by the authors.^18^ We suggest that mycobiome involvement in HS might be sufficient to explain why the use of antibiotics commonly results in treatment failure, as also evidenced in the 2 patients described here. Additionally, skin shrinkage following weight loss might be sufficient to increase the density of sebaceous glands that secrete sebum, thereby increasing the concentration of sebum per area of skin and available as a source of nutrients necessary to restore the normal skin microbiome and reverse the severity of HS. Short-chain fatty acids have also been shown to increase leptin secretion by adipose cells.^19^ Leptin is also known to stimulate sebaceous glands to secrete sebum.^20^

Our current clinical practice is for patients referred to our service to demonstrate their motivation and commitment to sustaining weight loss by first losing 5% of their body weight using the “No-P template” before being considered for IGB insertion. As exemplified by patient 3, we encourage our patients to continue the “No-P template” while their IGB remains *in situ*. Empirical experimentation to unambiguously verify that weight loss achieved using our dietary regimen designated the “No-P template” along with IGB insertion can cause the gut microbiome to normalize is now warranted. The gut-skin-microbiome axis is complex and not well understood. Although changes in the skin and, perhaps, gut micro/mycobiomes may play a role in the pathogenesis of HS, it is far from clear if dysbiosis is a primary event in the etiology of HS or occurs as a result of disease progression. Experimental validation to facilitate this distinction is also now requisite.

## Conflicts of interest

Dr Oben is a Consultant to Apollo Endosurgery: this case series was not funded by Apollo.

## References

[1] Shalom G., Freud T., Harman-Boehm I., Polishchuk I., Cohen A.D. (2015). Hidradenitis suppurativa, and metabolic syndrome: a comparative cross-sectional study of 3207 patients. Br J Dermatol.

[2] Hurley H.J., Roenigk R.K., Roenigk H.H. (1989). *Dermatologic Surgery*.

[3] Hirt P.A., Castillo D.E., Yosipovitch G., Keri J.E. (2019). Skin changes in the obese patient. J Am Acad Dermatol.

[4] Bettoli V., Naldi L., Cazzaniga S. (2016). Overweight, diabetes and disease duration influence clinical severity in hidradenitis suppurativa-acne inversa: evidence from the national Italian registry. Br J Dermatol.

[5] Alikhan A., Lynch P.J., Eisen D.B. (2009). Hidradenitis suppurativa: a comprehensive review. J Am Acad Dermatol.

[6] Nazary M., van der Zee H.H., Prens E.P., Folkerts G., Boer J. (2011). Pathogenesis and pharmacotherapy of hidradenitis suppurativa. Eur J Pharmacol.

[7] Pink A., Anzengruber F., Navarini A.A. (2018). Acne and hidradenitis suppurativa. Br J Dermatol.

[8] Kromann C.B., Deckers I.E., Esmann S., Boer J., Prens E.P., Jemec G.B. (2014). Risk factors, clinical course and long-term prognosis in hidradenitis suppurativa: a cross-sectional study. Br J Dermatol.

[9] Sivanand A., Gulliver W.P., Josan C.K., Alhusayen R., Fleming P.J. (2020). Weight loss and dietary interventions for hidradenitis suppurativa: a systematic review. J Cutan Med Surg.

[10] Nguyen V., Li J., Gan J. (2017). Outcomes following serial intragastric balloon therapy for obesity and nonalcoholic fatty liver disease in a single centre. Can J Gastroenterol Hepatol.

[11] Shai I., Schwarzfuchs D., Henkin Y. (2008). Weight loss with a low-carbohydrate, Mediterranean, or low-fat diet. N Engl J Med.

[12] Nguyen V., Mandour M.O., Bianco S.D. (2022). Metabolic endoscopy with intra-gastric balloon, improves obesity related hepatic steatosis indices, with changes in gut microbiota. J Obes Weight Loss Ther.

[13] McCarthy S., Barrett M., Kirthi S. (2022). Altered skin and gut microbiome in hidradenitis suppurativa. J Invest Dermatol.

[14] den Besten G., Bleeker A., Gerding A. (2015). Short-chain fatty acids protect against high-fat diet–induced obesity via a PPARγ-dependent switch from lipogenesis to fat oxidation. Diabetes.

[15] Kamp S., Fiehn A.M., Stenderup K. (2011). Hidradenitis suppurativa: a disease of the absent sebaceous gland? Sebaceous gland number and volume are significantly reduced in uninvolved hair follicles from patients with hidradenitis suppurativa. Br J Dermatol.

[16] Wark K.J.L., Cains G.D. (2021). The microbiome in hidradenitis suppurativa: a review. Dermatol Ther (Heidelb).

[17] Jiang S.W., Whitley M.J., Mariottoni P., Jaleel T., MacLeod A.S. (2021). Hidradenitis suppurativa: host-microbe and immune pathogenesis underlie important future directions. JID Innov.

[18] Fincher J.A., Jones D.R., Korte A.R. (2019). Mass spectrometry imaging of lipids in human skin disease model hidradenitis suppurativa by laser desorption ionization from silicon nanopost arrays. Sci Rep.

[19] Xiong Y., Miyamoto N., Shibata K. (2004). Short-chain fatty acids stimulate leptin production in adipocytes through the G protein-coupled receptor GPR41. Proc Natl Acad Sci U S A.

[20] Melnik B.C. (2016). Is sebocyte-derived leptin the missing link between hyperseborrhea, ductal hypoxia, inflammation and comedogenesis in acne vulgaris?. Exp Dermatol.

